# Hypercalcemia of malignancy in metastatic esophageal squamous cell carcinoma with simultaneous PTHrP and calcitriol overproduction: A case report with literature review

**DOI:** 10.1016/j.amsu.2021.102667

**Published:** 2021-08-05

**Authors:** Runbo Song, Yadav Bijay, Sophia H. Rizk, Shanjin Cao

**Affiliations:** a Department of General Surgery, Shijiazhuang 3rd Hospital, Shijiazhuang, Hebei Province, China; b Department of Hospitalist, St. Anne's Hospital, Fall River, MA, USA; c Department of Hematology Oncology, Charlton Memorial Hospital, Fall River, MA, USA; d Prima CARE, P.C., Fall River, MA, USA

**Keywords:** Hypercalcemia of malignancy, Parathyroid hormone-related peptide, 25-Hydroxyvitamin D, 1,25-Dihydroxyvitamin D, Calcitriol, Esophageal squamous cell carcinoma

## Abstract

**Introduction and importance:**

hypercalcemia of malignancy is a severe complication of malignancy and associated with poor prognosis. Four mechanisms are implicated in this metabolic disorder, including excess parathyroid-related peptide secretion, focal osteolysis secondary to bone metastasis or multiple myeloma, excess calcitriol production, and ectopic parathyroid hormone production. Humoral hypercalcemia of malignancy secondary to isolated PTHrP or calcitriol overproduction is known; however, hypercalcemia of malignancy due to simultaneous PTHrP and calcitriol overproduction is less well known.

**Case presentation:**

we report a case of a 63-year-old male who was diagnosed with poorly differentiated esophageal squamous cell carcinoma with simultaneous PTHrP and calcitriol overproduction.

**Clinical discussion:**

while hypercalcemia of malignancy secondary to simultaneous PTHrP and calcitriol secretion has been reported in other solid cancers, this is the first case of humoral hypercalcemia of malignancy secondary to simultaneous PTHrP and calcitriol secretion associated with esophageal cancer. This phenomenon deserves increased recognition as it has both diagnostic and therapeutic consequences. We discuss the current testing algorithm and its limitations in determining the etiology of hypercalcemia of malignancy since it may miss the diagnosis of simultaneous PTHrP and calcitriol production.

**Conclusion:**

we propose a revised testing algorithm for hypercalcemia of malignancy, which may improve the identification of simultaneous overproduction of PTHrP and calcitriol. This new algorithm can better characterize the mechanisms of hypercalcemia of malignancy and more appropriately guide treatment.

## Highlights

• Hypercalcemia of malignancy with simultaneous PTHrP and calcitriol production merits better recognition for both diagnostic and therapeutic purposes.
• A case of hypercalcemia of metastatic esophageal squamous cell carcinoma with simultaneous PTHrP and calcitriol is reported.
• The current testing algorithm and its limitations of hypercalcemia of malignancy is discussed, and a revised one is proposed.

## 1 Introduction

Hypercalcemia is a severe complication of malignancy, occurring in 0.20–0.67% of cases, and is associated with poor prognosis [1,2]. Hypercalcemia of malignancy is attributed to four major mechanisms [3,4]: excess parathyroid-related peptide (PTHrP) secretion, focal osteolysis secondary to cancer bone metastasis or multiple myeloma, excess 1,25-dihydroxyvitamin D (calcitriol) production, and rarely ectopic parathyroid hormone production [5]. Hormonal hypercalcemia of malignancy secondary to isolated PTHrP or calcitriol overproduction is well-known. However, hypercalcemia of malignancy due to simultaneous PTHrP and calcitriol overproduction lacks widespread recognition. This report describes a patient with esophageal squamous cell carcinoma with pleural metastasis who was found to have hypercalcemia in the setting of simultaneous PTHrP and calcitriol overproduction. This case gives us an opportunity to discuss the challenges of the current workup algorithm and its limitations in determining the etiology of hypercalcemia of malignancy, especially when there is simultaneous PTHrP and calcitriol production.

## 2 Case presentation

A 63-year-old male presented by ambulance with 3 months of dysphagia with solid and liquid food, 7 days of right lower rib cage pleuritic chest pain, and 5 days of constipation associated with nausea, vomiting, poor oral intake, and generalized weakness.

The patient has a history of moderately differentiated mid-esophageal invasive squamous cell carcinoma diagnosed nine months prior to admission, and received chemo- and radiation therapy for 7 weeks; patient refused surgery. Follow-up positron emission tomography-computed tomography and esophagogastroduodenoscopy (EGD) with biopsy 5 months prior to admission were negative for esophageal cancer.

His chronic medical problems included essential hypertension, mixed hyperlipidemia, gastroesophageal reflux disease, and right pre-auricular stage II (T2, N0, M0) skin basal cell carcinoma treated with radiation therapy 2 years prior to admission, currently in remission. He had a personal history of tobacco use and quit 9 months prior to admission. Home medications included atorvastatin, fenofibrate, morphine sulfate ER, oxycodone/acetaminophen, and docusate sodium.

The serum calcium was 9.4 mg/dL at the time of diagnosis of esophageal cancer 9 months prior to admission, and was between 9.1 and 10.4 mg/dL until this admission. The serum laboratory results on admission are listed in Table 1. The corrected calcium for albumin was 17.1 + 0.8 (4–3.7) = 17.3 mg/dL. In the setting of low PTH and high PTHrP, his hypercalcemia was consistent with hypercalcemia of malignancy.

**Table 1:** Serum laboratory results.

Laboratory test (Unit)	Result	Reference value
Calcium (mg/dL)	17.1	8.7–10.5
Phosphorus (mg/dL)	1.1	2.5–4.5
Magnesium (mg/dL)	1.1	1.8–2.5
Albumin (d/dL)	3.7	4.0–5.0
PTH intact (pg/mL)	<2	15–65
PTHrP (pg/mL)	80	14–27

The patient was treated with IV hydration with 0.9% sodium chloride, zoledronic acid 4 mg IV once, and calcitonin salmon 400 units subcutaneous q12h for 3 days. Serial serum calcium on admission day 1 was 13.5 mg/dL; day 2, 12.5 mg/dL; day 3, 11.7 mg/dL; day 4, 10.4 mg/dL, and day 5, 10.2 mg/dL.

For further workup of hypercalcemia and hypophosphatemia, 25-hydroxyvitamin D [25(OH)D] was measured and found to be 15 ng/mL (ref., ≥30 ng/mL). Calcitriol was tested and found to be 138 pg/mL (ref., 18–72 pg/mL). Because calcitriol was elevated, vitamin D supplementation was not pursued given the concern for inducing persistent hypercalcemia, despite the low 25(OH)D.

During investigation of the etiology of hypercalcemia of malignancy, CT chest with contrast revealed a large right pleural effusion, and multiple pleural and ipsilateral diaphragmatic coalescent soft tissue masses, which were new compared to the prior CT chest examinations. MRI brain with IV contrast revealed nonspecific white matter abnormalities and no brain metastasis. CT neck soft tissue and abdomen and pelvis with contrast revealed no esophageal mass or thickening; there was mild hepatic steatosis but no hepatic, pancreatic, or adrenal metastasis, and no lymphadenopathy. A double contrast barium swallow test was performed and showed the esophagus was normal in course, motility, and caliber without constricting lesions. A repeat EGD with biopsy showed a mid-esophageal ulcer with reactive squamous mucosa and no carcinoma. These findings were consistent with post-radiation esophagitis with a shallow esophageal ulcer and no in-situ esophageal carcinoma recurrence. The patient was started on a proton pump inhibitor (PPI) and modified dysphagia diet, resulting in improvement in symptoms.

An ultrasound-guided thoracentesis was performed for his right pleural effusion and pleural mass, and 1,750 mL of serous fluid was drained. Chemistry study showed an exudative effusion by Light's criteria; cytology revealed reactive mesothelial cells, acute and chronic inflammatory cell and histiocytes, and no malignant cells. One month later, a video-assisted thoracoscopy with pleural biopsy was performed. The pathology revealed poorly differentiated squamous cell carcinoma with immunostains positive for AE1/AE3, p40, p63, and MOC 31, and negative for calretinin, TTF1, and CK7. Taken together, the patient was diagnosed with esophageal squamous cell carcinoma with right pleural metastasis. An indwelling pleural catheter was inserted for recurrent right pleural effusions requiring weekly drainage.

Upon discharge, patient followed up with oncology via telemedicine and home visiting nurse for hospice care. Due to the poor prognosis, no feeding tube was inserted. Three months later patient passed away peacefully at home.

This work has been reported in line with the SCARE 2020 criteria [6].

## 3 Discussion

There exist four mechanisms accounting for hypercalcemia of malignancy, as listed above [3,4]. An individual patient, however, may have more than one mechanism involved. In this case, the patient's hypercalcemia can be attributed to simultaneous PTHrP and calcitriol overproduction. The patient also has hypophosphatemia, which is likely due to the effect of PTHrP increasing renal calcium reabsorption and phosphorus excretion [3]. Although hypophosphatemia can increase the conversion of 25(OH)D to calcitriol, hypercalcemia by contrast would decrease this conversion. Thus, the increased calcitriol is less likely due to hypophosphatemia in this case.

Hypercalcemia of malignancy due to simultaneous overproduction of PTHrP and calcitriol is thought to be rare [7]. This phenomenon has been reported mostly in case reports in lung squamous cell cancer [8], colorectal squamous cancer [9], skin squamous cancer [10], renal clear cell carcinoma [11], ovarian clear cell carcinoma [12], and seminoma [13]; there is also an association with other cancers including breast cancer, head and neck squamous cell cancer, bladder cancer, pancreatic cancer, sarcoma, neuroendocrine tumor, prostate cancer, and melanoma [7]. To our knowledge, no prior case report has described this phenomenon in esophageal cancer.

Chukir T et al. (2020) conducted a single institution retrospective study and found that 45/101 (45%) cases of hypercalcemia of malignancy in solid tumors had elevated calcitriol, of which 76% had elevated PTHrP, meaning 24.2% of solid tumor patients had simultaneous PTHrP and calcitriol overproduction [7]. This study suggests the incidence of simultaneous secretion of PTHrP and calcitriol in hypercalcemia of malignancy is higher than previously thought.

**Fig. 1:**
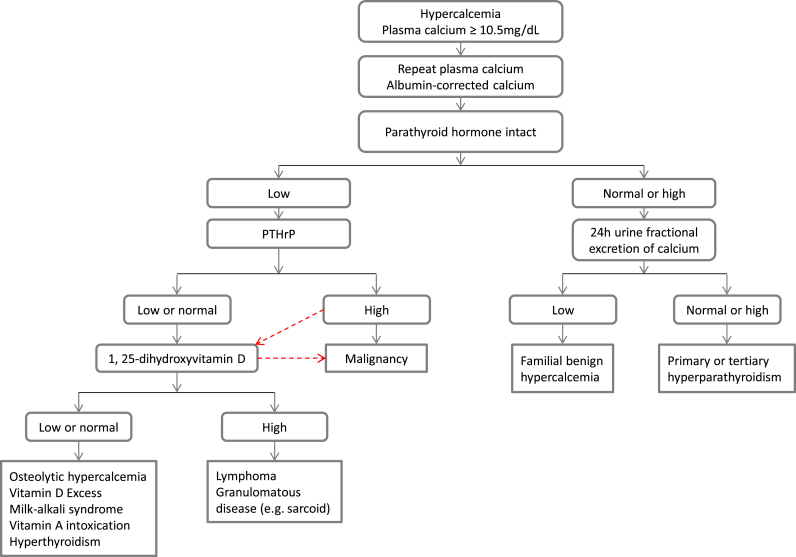
The current diagnostic algorithm of hypercalcemia of malignancy. PTHrP, parathyroid hormone-related peptide.

The current diagnostic algorithm of hypercalcemia [3] (Fig. 1) starts with PTH, which divides patients into two groups: one group with normal or high PTH, usually due to primary hyperparathyroidism or familial hypocalciuric hypercalcemia (FHH); and the other group with low PTH, usually due to malignancy or other endocrinopathies. The next step in the diagnostic workup is PTHrP. If PTHrP is high then generally the diagnosis of hypercalcemia of malignancy is made. If PTHrP is low or normal, then further testing of calcitriol is pursued. High calcitriol is suggestive of lymphoma or granulomatous disease; low calcitriol is suggestive of osteolytic hypercalcemia such as cancer bone metastasis, multiple myeloma, or other etiologies. As can be seen in this algorithm, the diagnostic pathways of elevated PTHrP and calcitriol are in parallel but do not intersect (red arrows in Fig. 1). This lack of crosstalk is likely the reason why the diagnosis of hypercalcemia of malignancy with simultaneous PTHrP and calcitriol overproduction can be missed by the current testing algorithm.

**Fig. 2:**
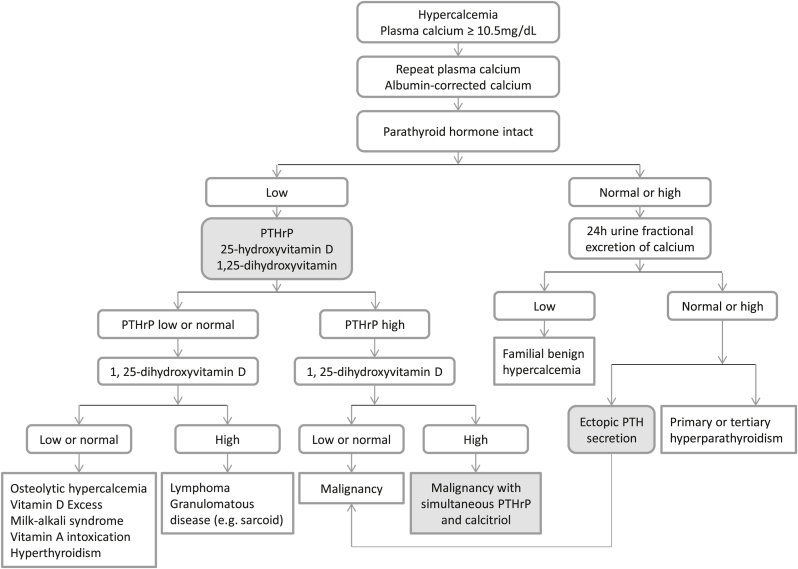
A proposed diagnostic algorithm of hypercalcemia of malignancy. PTHrP, parathyroid hormone-related peptide.

Goldner, W (2016) proposed that the initial laboratory tests for hypercalcemia of malignancy should include PTH, PTHrP, 25(OH)D, and calcitriol [14]. Integration of this more comprehensive diagnostic approach (Fig. 2) can overcome the limitations of the current testing algorithm (Fig. 1) for hypercalcemia of malignancy. This modification is necessary and useful for several reasons. Firstly, for diagnostic purposes, this new algorithm prevents missing hypercalcemia of malignancy due to simultaneous PTHrP and calcitriol overproduction. Secondly, recognizing this simultaneous PTHrP and calcitriol overproduction has treatment benefits. Routine treatment for hypercalcemia involves IV fluid therapy, furosemide (once adequately hydrated), calcitonin, and zoledronic acid. However, in the setting of concurrent calcitriol elevation, glucocorticoids may be needed to suppress 1α-hydroxylase activity [8,11,13]. Chukir T et al. (2020) showed that hypercalcemia of malignancy with elevated PTHrP and elevated calcitriol levels generally did not respond completely to antiresorptive treatment alone, whereas most patients with isolated elevated PTHrP without calcitriol elevation responded well to antiresorptive treatment alone [7]. Of note, in this case, glucocorticoids treatment was not initiated given serum calcium normalized with antiresorptive alone. Thirdly, if only 25-hydroxyvitamin D was tested, which was low in this case, vitamin D supplementation may have been started to improve bone health in the setting of hypophosphatemia. However, vitamin D supplementation in the setting of elevated calcitriol can lead to vitamin D toxicity and persistent hypercalcemia. Lastly, to account for all four mechanisms of hypercalcemia of malignancy [3,4], this new algorithm also includes ectopic secretion of PTH in malignancy, though it is rare [5] (Fig. 2).

It is important to note that in malignancy, the overproduction of PTHrP and calcitriol are two separate processes. PTH has the physiologic function of increasing kidney 1α-hydroxylase activity and thus increases calcitriol production. Despite its structural similarity to PTH, PTHrP has minimal or no effect in increasing kidney calcitriol production [3,15,16]. The comparison of the physiologic function of PTH and PTHrP on target organs has been summarized in a previous publication [3]. Therefore, while increased calcitriol in malignancy occurs through a mechanism of increased 1α-hydroxylase, this process is independent of increased PTHrP.

## 4 Conclusion

We present a case of hypercalcemia of malignancy in metastatic esophageal squamous cell carcinoma with simultaneous overproduction of PTHrP and calcitriol. This phenomenon deserves increased recognition as it has both diagnostic and therapeutic values. This case highlights the limitations of the current testing algorithm of hypercalcemia of malignancy. We propose a modified testing algorithm to help identify simultaneous overproduction of PTHrP and calcitriol. This improved characterization of the mechanisms of hypercalcemia of malignancy can guide more appropriate treatment.

## Provenance and peer review

Not commissioned, externally peer-reviewed.

## Ethical approval

Not required.

## Sources of funding

None.

## Author contribution

Shanjin Cao and Sophia H. Rizk collected patient's date. Runbo Song, Yadav Bijay, Sophia H. Rizk, and Shanjin Cao conducted the literature review and wrote the manuscript. All authors approved the final version of this paper.

## Consent

Written informed consent was obtained from the patient for publication of this case report and accompanying images. A copy of the written consent is available for review by the Editor-in-Chief of this journal on request.

## Registration of Research Studies

N/A.

1. Name of the registry: Not applicable.

2. Unique Identifying number or registration ID:

3. Hyperlink to your specific registration (must be publicly accessible and will be checked):

## Guarantor

Shanjin Cao.

## Declaration of competing interest

The authors have no conflict of interest to declare.
